# Healthy Campus Trial: a multiphase optimization strategy (MOST) fully factorial trial to optimize the smartphone cognitive behavioral therapy (CBT) app for mental health promotion among university students: study protocol for a randomized controlled trial

**DOI:** 10.1186/s13063-018-2719-z

**Published:** 2018-07-04

**Authors:** Teruhisa Uwatoko, Yan Luo, Masatsugu Sakata, Daisuke Kobayashi, Yu Sakagami, Kazumi Takemoto, Linda M. Collins, Ed Watkins, Steven D. Hollon, James Wason, Hisashi Noma, Masaru Horikoshi, Takashi Kawamura, Taku Iwami, Toshi A. Furukawa

**Affiliations:** 1 Kyoto University Health Service, Yoshida-Honmachi, Sakyo-ku, Kyoto, 606-8501 Japan; 2 Deparment of Health Promotion and Human Behavior, Kyoto University Graduate School of Medicine / School of Public Health, Yoshida Konoe-cho, Sakyo-ku, Kyoto, Japan; 3 The Methodology Center and Department of Human Development & Family Studies, The Pennsylvania State University, University Park, PA USA; 4 School of Psychology, University of Exeter, Exeter, UK; 5 Department of Psychology, Vanderbilt University, Nashville, TN USA; 6 MRC Biostatistics Unit, University of Cambridge, Cambridge, UK; 7 Institute of Statistical Mathematics, Tokyo, Japan; 8 Center for Cognitive Behavior Therapy and Research, National Center of Neurology and Psychiatry, Kodaira, Japan

**Keywords:** Cognitive behavioral therapy, Smartphone, Depression, MOST, Factorial, University students

## Abstract

**Background:**

Youth in general and college life in particular are characterized by new educational, vocational, and interpersonal challenges, opportunities, and substantial stress. It is estimated that 30–50% of university students meet criteria for some mental disorder, especially depression, in any given year. The university has traditionally provided many channels to promote students’ mental health, but until now only a minority have sought such help, possibly owing to lack of time and/or to stigma related to mental illness. Smartphone-delivered cognitive behavioral therapy (CBT) shows promise for its accessibility and effectiveness. However, its most effective components and for whom it is more (or less) effective are not known.

**Methods/design:**

Based on the multiphase optimization strategy framework, this study is a parallel-group, multicenter, open, fully factorial trial examining five smartphone-delivered CBT components (self-monitoring, cognitive restructuring, behavioral activation, assertion training, and problem solving) among university students with elevated distress, defined as scoring 5 or more on the Patient Health Questionnaire-9 (PHQ-9). The primary outcome is change in PHQ-9 scores from baseline to week 8. We will estimate specific efficacy of the five components and their interactions through the mixed-effects repeated-measures analysis and propose the most effective and efficacious combinations of components. Effect modification by selected baseline characteristics will be examined in exploratory analyses.

**Discussion:**

The highly efficient experimental design will allow identification of the most effective components and the most efficient combinations thereof among the five components of smartphone CBT for university students. Pragmatically, the findings will help make the most efficacious CBT package accessible to a large number of distressed university students at reduced cost; theoretically, they will shed light on the underlying mechanisms of CBT and help further advance CBT for depression.

**Trial registration:**

UMIN, CTR-000031307. Registered on February 14, 2018.

## Background

It is estimated that between 30% and 50% of university students meet criteria for at least one mental disorder in any given year [1, 2]. Mental disorders, when untreated, can have significant consequences: they have been shown to lead to lowered academic performance [3], increased dropout from university before completion [4, 5], and impairment in social relationships [6]. They are also often associated with various comorbidities, including suicidality [7], substance use [8], and subsequent development of a range of mental disorders. Most lifetime mental disorders have their first onset before age 24 [9]; they account for nearly half of disease burden for young adults globally [10].

For many, young adulthood is a period of greater educational opportunities, prospects for employment, and development of personal relationships. Given the breadth of new experiences, opportunities, and challenges faced by university students, it is not surprising that college life is characterized by substantial stress. College campuses have accordingly been providing many channels through which they can have a positive effect on the mental health of their youth. Unfortunately, however, surveys repeatedly show that only a minority of distressed university students seek professional help, possibly due in part to lack of time and also to stigma related to mental health [1, 11].

Clearly, we need new approaches and strategies [12]. Medications are not effective and are possibly harmful for depression in youth [13, 14], especially when depression is mild and subthreshold [15]. By contrast, there is growing evidence that computer-delivered and internet-based psychological interventions, in particular cognitive behavioral therapy (CBT), may alleviate depression and anxiety in youth [16] and in university students [17]. Digital interventions increase accessibility and decrease cost and thus can meet high-scale demands and ensure quality in delivery [18]. The young generation may find them particularly attractive and easy to access, thereby surmounting their many barriers to help-seeking.

Psychological interventions, however, are complex and consist of multiple components in variable combinations [19]. For example, CBT may typically include components such as psychoeducation (PE), self-monitoring (SM), cognitive restructuring (CR), behavioral activation (BA), assertion training (AT), problem solving (PS), relaxation, and mindfulness, among others. But traditional randomized controlled trials (RCTs) have only examined the effectiveness of various combinations of these as a package. It is then no wonder that some studies of such broadly conceived CBT programs consisting of different components have produced conflicting results in terms of their effectiveness [16, 17]. It remains to be seen which of the various cognitive and behavioral skills are effective in alleviating depression and anxiety among university students, and for whom they may be particularly fit or unfit.

The multiphase optimization strategy (MOST) is an innovative approach rooted in engineering and behavioral science that provides a principled and comprehensive framework for selecting individual intervention components out of multicomponent interventions [20, 21]. It consists of three stages: (1) preparation to conduct an optimization trial, (2) optimization to reveal what constitutes an optimized intervention, and (3) evaluation of the optimized intervention relative to an established intervention in an RCT.

In this study, we use the three cognitive behavioral components for SM, CR, and BA that were included in an internet CBT program with demonstrated effectiveness [18] and two similarly constructed components for AT and PS. This study represents the optimization phase according to MOST and uses the fully factorial design that allows estimation of main effects of individual components and their interaction effects. In this design, the effect of a component is estimated by comparing the mean of all combinations including that component against the mean of all other combinations not including that component. Because both the former and the latter sets of combinations have equal credibility and nonspecific supportive elements, this design allows estimation of effects specific to that component and can uniquely overcome the issue of specificity in psychotherapy research [22], which has been unable to find a nonspecific control equal to pill placebo in drug research [23, 24] and has had formidable difficulties in elucidating effects specific to a certain intervention over and above nonspecific psychotherapies. A further challenge associated with multicomponent interventions is matching particular components with individual characteristics: Some components may be particularly fit or unfit for certain subgroups of participants. There have been several attempts to tailor or personalize psychotherapies to match individual characteristics [25, 26]. Smartphone CBT offers a unique opportunity to examine effect modification by individual characteristics because it enables recruitment of the large sample of participants that would be required to examine the issue.

The purpose of the Healthy Campus Trial is therefore to examine specific efficacy of CBT components, thereby determining the most effective and efficient combination for university students and exploring the matching of the selected components with individual characteristics. Given the long-lasting and potentially serious consequences of psychological distress, the study will have two analytical phases: analysis 1 (stress reduction study) and analysis 2 (prevention study).

This protocol has been written in accordance with the Standard Protocol Items: Recommendations for Interventional Trials (SPIRIT) guideline [27] and with the Japanese Ethical Guidelines for Medical and Health Research Involving Human Subjects (December 22, 2014) and its guidance (revised May 29, 2017). This report is based on protocol version 1.0.1, approved on February 24, 2018, by Kyoto University Graduate School of Medicine Ethics Committee.

## Methods/design

### Objectives

The primary objectives of analysis 1 are twofold:

1. To find if each component has specific efficacy in terms of short-term (2 months) alleviation of psychological distress, including depression and anxiety. The biggest strength of the factorial design is that the examination of each component as a main effect can reveal the specific efficacy of that component in comparison with not receiving that component.
2. To find the most efficacious components and the most efficient combinations thereof out of the five typical CBT skills for the university students. The identified combinations should be efficient in terms of the time and effort required of the students and the costs incurred by the university.

The trial is also designed to answer the following ancillary questions:

3. To conduct exploratory analyses to find the best matches between students’ baseline characteristics and the CBT components so that we can offer personalized and improved services in the future.
4. To examine the ordering effect of BA and CR, the two most basic components of CBT.
5. To explore the process variables in the efficacy of the smartphone CBT.
6. To test for enduring effects of the 2-month stress reduction at 12 months.

The target population of analysis 1 is those students with elevated distress at baseline, defined as scoring 5 or more on the Patient Health Questionnaire-9 (PHQ-9) [28].

The primary objectives of analysis 2 are as follows:

1. To examine the long-term effect (12 months) of the most efficacious and efficient combination(s) in preventing future occurrences of new depressive episodes.
2. To examine the ordering effect of BA and CR and to explore the process variables in the efficacy of the smartphone CBT in preventing future occurrences of new depressive episodes.

The target population of analysis 2 is those without a major depressive episode (MDE) as ascertained by Composite International Diagnostic Interview (CIDI) at baseline.

### Trial design

This trial is a parallel-group, multicenter, open, stratified block randomized, fully factorial trial of five experimental components of SM, CR, BA, AT, and PS. Each component will be coded at two levels (+ 1 for presence and − 1 for absence).

### Participants

#### Study setting

This trial will take place at Kyoto University and several other universities in Japan. The collaborating universities will be recruited at the later stage of the study.

#### Eligibility criteria for study centers

Collaborating universities will be recruited from 4-year universities in Japan, taking into consideration their willingness to participate, their size, and other characteristics so that there will be more variability in the backgrounds of the participants.

#### Eligibility criteria for participants

Each participant must satisfy all of the following inclusion criteria and none of the exclusion criteria.

##### Inclusion criteria

1. University students enrolled in full-time undergraduate or graduate programs at the participating universities
  - Aged between 18 and 39 at time of enrollment. The upper age limit of 39 was adopted in order to secure homogeneity of the participants within adolescence to young adulthood.
  - Of either sex
2. They must have their own smartphone, either an Apple iPhone or Android device.
3. They must provide written informed consent to participate in this study after full disclosure of the contents and procedures of the study.
4. They must have completed the PE component within 2 weeks after providing their consent.

##### Exclusion criteria

1. Being unable to understand written Japanese
2. Currently receiving professional treatment for mental health problems
3. Scoring 10 or more on PHQ-9 at screening

##### Eligibility criteria for study personnel

Encouragement emails will be handled by personnel qualified in health care, such as physicians, nurses, and clinical psychologists.

### Interventions

**Fig. 1:**
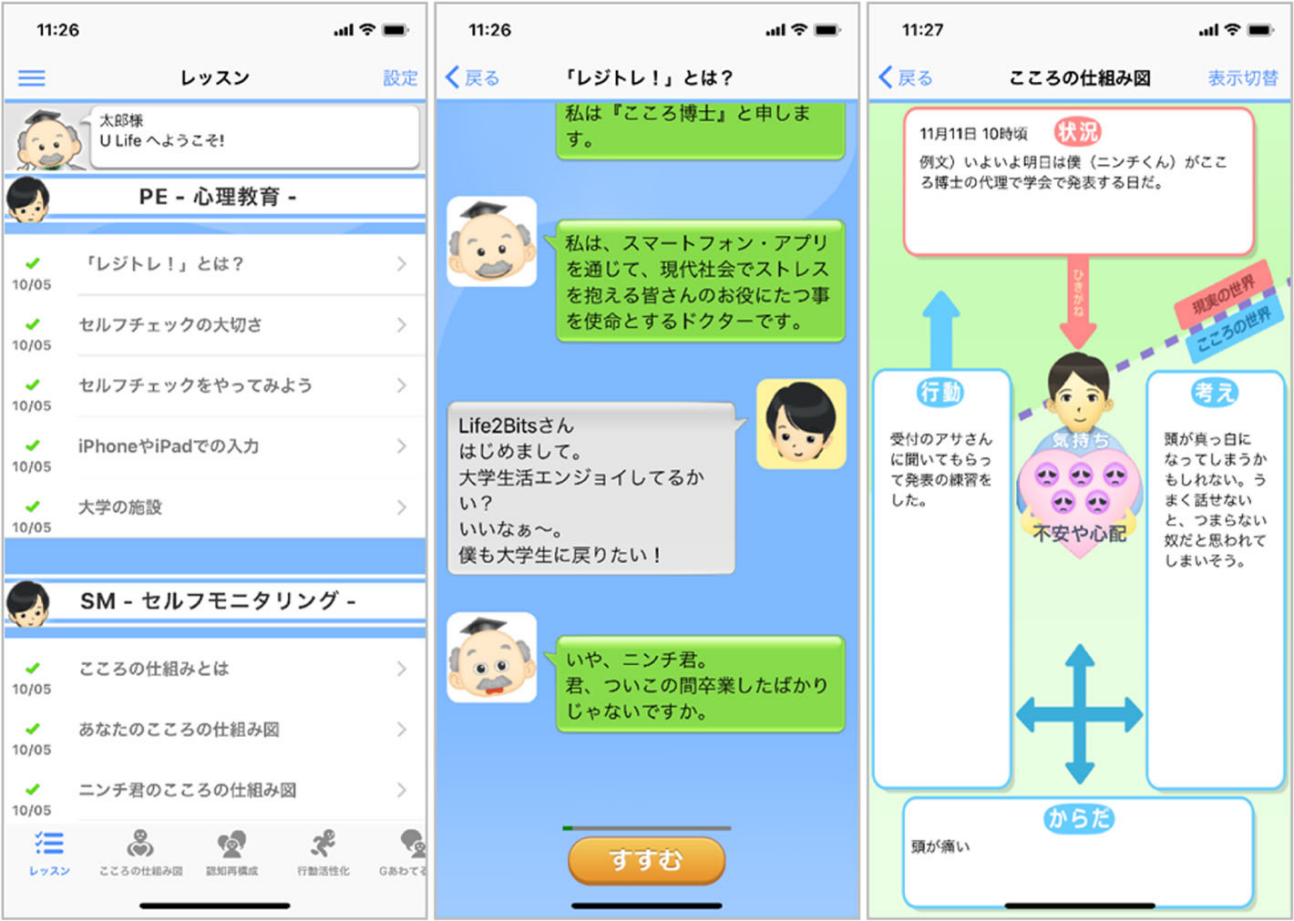
Screenshots from the cognitive behavioral therapy smartphone app

Figure 1 shows the screenshots from the smartphone app. The components of the smartphone CBT are as follows:

- **PE** (psychoeducation) consists of didactic materials about psychological stress, emphasizes importance of self-checks of one’s own emotional states, and provides information about campus resources for psychological help.
- **SM** (self-monitoring) consists of PE of the CBT model in the form of a mind map. The participants learn how to monitor their reactions to situations in terms of feelings, thoughts, body reactions, and behaviors and describe them in mind maps. They will be asked to fill in at least one mind map from their daily life before they can proceed to the next lesson. They are then free to complete as many mind maps as they can during the intervention.
- **CR** (cognitive restructuring) consists of PE of skills of cognitive restructuring and a worksheet of finding alternative thoughts for a recent stressful situation. In order to help the participants broaden their thoughts, CR provides four tools, each of which guides them to alternative thoughts through interaction with the characters.
- **BA** (behavioral activation) consists of PE of the importance of pleasurable activities according to the principle “When your body moves, so does your mind.” It provides a worksheet of a personal experiment to test a new activity and also a gamified “action marathon” to promote such personal experiments.
- **AT** (assertion training) consists of PE of assertive communication in contrast to aggressive or passive communication. The participants learn how to express their true feelings and wishes without hurting others or sacrificing themselves.
- **PS** (structured problem solving) teaches the participants how to break down the issue at hand, to specify a concrete and achievable objective for it, to brainstorm possible solutions, to compare their advantages and disadvantages, and finally to choose the most desirable action and act on it. A worksheet to guide the participants through this process is provided.

Each component will consist of a lesson that is supposed to take 1 week to complete. The participants can move to the next lesson only after 1 week has passed and after they have completed one worksheet. We have demonstrated the efficacy of a smartphone CBT package containing the SM, BA, and CR components among patients with antidepressant-resistant depression in an RCT [18]. This study found that it took the participants, on average, 10.8 (SD, 4.2) days to complete one lesson; because the maximum number of components or lessons that participants undertake after PE is five, we set the length of the intervention phase of the trial to 8 weeks.

PE constitutes the core of the intervention, and all participants will receive this component. After completing PE, the participants will be randomly allocated to one of the combinations on the basis of presence/absence of the remaining five components (Table 1). In order to enable examination of the ordering effects between CR and BA, we doubled the number of combinations to 2^5^ × 2 = 64. AT and PS are placed before or after SM or CR or BA in equal proportions among these 64 combinations in order to counterbalance the anticipated likelihood that fewer participants will complete later parts of the intervention. All participants have full access to treatment as usual, inside and outside the campus.

**Table 1:** Combinations of cognitive and behavioral components of cognitive behavioral therapy smartphone app

	PE	SM	CR	BA	AT	PS	Sequence of administration								No.
C1	1	1	1	1	1	1	PE	AT	PS	SM	CR	BA			17
C2	1	1	1	1	1	1	PE			SM	BA	CR	AT	PS	17
C3	1	1	1	1	1	−1	PE			SM	CR	BA	AT		17
C4	1	1	1	1	1	−1	PE	AT		SM	BA	CR			17
C5	1	1	1	1	−1	1	PE		PS	SM	CR	BA			17
C6	1	1	1	1	−1	1	PE			SM	BA	CR		PS	17
C7	1	1	1	1	−1	−1	PE			SM	CR	BA			17
C8	1	1	1	1	−1	−1	PE			SM	BA	CR			17
C9	1	1	1	−1	1	1	PE			SM	CR		AT	PS	17
C10	1	1	1	−1	1	1	PE	AT	PS	SM	CR				17
C11	1	1	1	−1	1	−1	PE			SM	CR		AT		17
C12	1	1	1	−1	1	−1	PE	AT		SM	CR				17
C13	1	1	1	−1	−1	1	PE			SM	CR			PS	17
C14	1	1	1	−1	−1	1	PE		PS	SM	CR				17
C15	1	1	1	−1	−1	−1	PE			SM	CR				17
C16	1	1	1	−1	−1	−1	PE			SM	CR				17
C17	1	1	−1	1	1	1	PE			SM		BA	AT	PS	17
C18	1	1	−1	1	1	1	PE	AT	PS	SM		BA			17
C19	1	1	−1	1	1	−1	PE			SM		BA	AT		17
C20	1	1	−1	1	1	−1	PE	AT		SM		BA			17
C21	1	1	−1	1	−1	1	PE			SM		BA		PS	17
C22	1	1	−1	1	−1	1	PE		PS	SM		BA			17
C23	1	1	−1	1	−1	−1	PE			SM		BA			17
C24	1	1	−1	1	−1	−1	PE			SM		BA			17
C25	1	1	−1	−1	1	1	PE			SM			AT	PS	17
C26	1	1	−1	−1	1	1	PE	AT	PS	SM					17
C27	1	1	−1	−1	1	−1	PE			SM			AT		17
C28	1	1	−1	−1	1	−1	PE	AT		SM					17
C29	1	1	−1	−1	−1	1	PE			SM				PS	17
C30	1	1	−1	−1	−1	1	PE		PS	SM					17
C31	1	1	−1	−1	−1	−1	PE			SM					17
C32	1	1	−1	−1	−1	−1	PE			SM					17
C33	1	−1	1	1	1	1	PE				CR	BA	AT	PS	17
C34	1	−1	1	1	1	1	PE	AT	PS		BA	CR			17
C35	1	−1	1	1	1	−1	PE	AT			CR	BA			17
C36	1	−1	1	1	1	−1	PE				BA	CR	AT		17
C37	1	−1	1	1	−1	1	PE				CR	BA		PS	17
C38	1	−1	1	1	−1	1	PE		PS		BA	CR			17
C39	1	−1	1	1	−1	−1	PE				CR	BA			17
C40	1	−1	1	1	−1	− 1	PE				BA	CR			17
C41	1	−1	1	−1	1	1	PE				CR		AT	PS	17
C42	1	−1	1	−1	1	1	PE	AT	PS		CR				17
C43	1	−1	1	−1	1	− 1	PE				CR		AT		17
C44	1	−1	1	−1	1	−1	PE	AT			CR				17
C45	1	−1	1	−1	−1	1	PE				CR			PS	17
C46	1	−1	1	−1	−1	1	PE		PS		CR				17
C47	1	−1	1	−1	−1	−1	PE				CR				17
C48	1	−1	1	−1	−1	−1	PE				CR				17
C49	1	−1	−1	1	1	1	PE					BA	AT	PS	17
C50	1	−1	−1	1	1	1	PE	AT	PS			BA			17
C51	1	−1	−1	1	1	−1	PE					BA	AT		17
C52	1	−1	−1	1	1	− 1	PE	AT				BA			17
C53	1	−1	−1	1	−1	1	PE					BA		PS	17
C54	1	−1	−1	1	−1	1	PE		PS			BA			17
C55	1	−1	−1	1	−1	− 1	PE					BA			17
C56	1	−1	−1	1	−1	− 1	PE					BA			17
C57	1	−1	−1	−1	1	1	PE						AT	PS	17
C58	1	−1	−1	−1	1	1	PE	AT	PS						17
C59	1	−1	−1	−1	1	−1	PE						AT		17
C60	1	−1	−1	−1	1	−1	PE	AT							17
C61	1	−1	−1	−1	− 1	1	PE							PS	17
C62	1	−1	−1	−1	− 1	1	PE		PS						17
C63	1	−1	−1	−1	− 1	−1	PE								17
C64	1	−1	−1	−1	− 1	-1	PE								17

*Abbreviations: AT* Assertion training, *BA* Behavioral activation, *CR* Cognitive restructuring, *PE* Psychoeducation, *PS* Problem solving, *SM* Self-monitoring

*Note*: 1 denotes presence and − 1 denotes absence of that component; blue cells denote the combination where the ordering of CR and BA is reversed

### Strategies to improve adherence to interventions and procedures for monitoring adherence

The participants will receive a semiautomated email to congratulate them for their progress in the program every week. The message is based on a template focusing on adherence and motivation but can be modified by a physician or a clinical psychologist on the study team. It can contain technical advice for the program, but the participant will be referred to the student health center for any psychological advice.

The adherence of the participants to the allocated intervention will be uploaded and recorded by the online server program. The study personnel will have access to the server and perform ongoing monitoring of adherence of the participants.

### Concomitant care and interventions that are permitted or prohibited during the trial

All the participants are allowed to receive their usual treatments, including services at the student health center and other professional psychiatric care. Any receipt of psychological or pharmacological care for a mental health problem will be recorded.

### Criteria for discontinuing or modifying the allocated interventions

The allocated intervention will be discontinued if any of the following criteria are met. The date and reason will be recorded. The follow-up assessments will be continued if the participant does not withdraw his or her consent to be assessed.

1. The participant withdraws from the intervention
2. The participant cannot continue the intervention owing to an adverse event
3. The study itself is discontinued
4. The steering committee judges that it is appropriate to discontinue the intervention.

### Anticipated risks and benefits for the participants

The interventions will consist of various amounts of PE and exercises in cognitive and behavioral skills for stress reduction to be conducted as self-help by the participants on their smartphones. The interventions will therefore be considered as “intervention with minimal invasiveness,” and no serious health risks are expected, except for possible psychological and time burden in going through the program and responding to the questionnaires. On the contrary, a meta-analysis of internet-based guided self-help programs show that participants have lower risk of deterioration in symptoms of depression during such interventions than control groups [29].

The possible psychological and time burdens will be fully disclosed and explained to the potential participants at the orientation meeting, and only those who have provided written informed consent will be recruited into the study. No insurance scheme is therefore planned.

However, possibility of serious adverse events (SAEs), whether related to the study participation or not, cannot be negated as would be normally would be expected of participants leading their university student lives. They will be closely monitored, and all participants will be provided standard care through the student health center at the university. Any expenses necessary for such services will be handled as usual. On the other hand, the participants can expect to learn about psychological stress and about how to maintain mental health through the program. But the extent of such benefits is the theme of this trial and is currently unknown.

### Preventing contaminations

Because the intervention takes place at the same university, there is some concern regarding contamination among the friends, each of whom is randomized to a different combination of components but who may discuss the components of their peers. We assume that such a possibility is minimal, however, because the intervention takes place on each participant’s own smartphone, which makes it hard to share the actual contents shown on the app. We will take extra precaution to prevent the contamination by explicitly instructing the participants not to discuss or exchange the apps to which each is assigned. Each participant will be inquired as to the degree of possible contamination at week 8, and these results will be reported to and monitored by data safety and monitoring committee.

### Measurements

#### Primary outcomes

The primary outcome of analysis 1 is self-administered PHQ-9 at week 8. The primary outcome of analysis 2 is incidence of a MDE as assessed by the MDE section of the computerized CIDI at week 52.

#### Secondary outcomes

The secondary outcomes are as follows:

1. PHQ-9 at weeks 1, 2, 3, 4, 5, 6, 7, 12, 16, 20, 24, 28, 32, 36, 40, 44, 48, and 52
2. Generalized Anxiety Disorder-7 (GAD-7) at weeks 4, 8, and 52
3. Cognitive and behavioral skills [30–35] at weeks 8 and 52
4. Presenteeism scale from the Health and Work Performance Questionnaire (HPQ) [36] at weeks 8 and 52
5. Use statistics of the smartphone app through the trial
6. Cointerventions (visits to a mental health professional, psychotropics, counseling/psychotherapy)

#### Background characteristics

The following background characteristics will be measured at baseline:

1. Demographics
  - Age
  - Sex
  - Course (doctoral/master/undergraduate) and class (1/2/3/4: if repeated or not)
  - Major
  - Marital status
  - Domicile (home/lodging)
  - Part-time employment (no/less than 2 h per week/2–10 h/more than 10 h)
  - Involvement in club activities (no/less than 2 h per week/2–10 h/more than 10 h)
  - History of psychological/psychiatric treatment
  - Smoking (none/10 cigarettes/20 cigarettes/30 or more cigarettes per day)
  - Drinking (none/less than 2 units per day/2 or more units per day/problem drinking)
  - Exercise (rarely/sometimes/daily)
  - Breakfast (rarely/sometimes/every day)
2. Personality
  - Short form of the Big Five scale (29 items) [37, 38]
  - Short form of the Autism Spectrum Quotient (ten items) [39]
3. Social support
  - Social Support Questionnaire (12 items) [40, 41]
4. Cognitive and behavioral skills
  - Self-monitoring (five items) [30]
  - Cognitive restructuring (six items) [31]
  - Behavioral activation: Behavioral Activation for Depression Scale-Short Form (eight items) [32, 33]
  - Assertiveness (seven items) [34]
  - Problem solving (six items) [35]
5. Clinical characteristics
  - PHQ-9 [28]
  - GAD-7 [42]
  - Major depression section of the computerized World Health Organization (WHO) CIDI [43, 44]
6. Function
  - Presenteeism scale from the HPQ [36]

### Measures

#### Personal Health Questionnaire-9 (PHQ-9)

PHQ-9 consists of the nine diagnostic criteria items for a MDE in the *Diagnostic and Statistical Manual of Mental Disorders, Fourth Edition* (DSM-IV), and the DSM-5 [28]. Each item is rated from 0 = *Not at all* through 3 = *Nearly every day*, with the total score ranging therefore between 0 and 27. The reliability and the validity of the original PHQ-9 and its Japanese version are well established [45, 46]. We used this scale successfully in our previous trial of the smartphone CBT [18].

#### Generalized Anxiety Disorder-7

GAD-7 was developed to measure the severity of generalized anxiety and consists of seven items representing nervousness, tension, and worrying [42]. Each item is rated from 0 = *Not at all* through 3 = *Nearly every day*, with the total score ranging therefore between 0 and 21. Its reliability and validity have been established [42]. The use of PHQ-9 and GAD-7 has been recommended as part of a standardized battery to measure health outcomes for depression and anxiety [47].

#### Big Five Scale of Personality Trait Adjectives

There are several validated scales to measure personality traits according to the five-factor model. We will use the short form of the Big Five Scale of Personality Trait Adjectives [37], commonly used in Japan. The reliability and validity of the short version have been ascertained [38]. Each of the five personality traits of neuroticism, extraversion, openness, agreeableness, and conscientiousness is measured with five to seven corresponding adjectives on a 5-point Likert scale from 0 = *Untrue of me* and 4 = *True of me*.

#### Autism Spectrum Quotient

The autistic trait of the participants will be measured with the short version of the Autism Spectrum Quotient, originally developed by Baron-Cohen et al. [48]. The reliability and validity of the Japanese version and its short from have been established [39].

#### Social Support Questionnaire

We will measure the size and quality of the students’ social support with the short form of the Social Support Questionnaire [40]. It measures the number of persons providing support and the satisfaction with such support in six domains. The reliability and validity of the original scale and its Japanese version have been satisfactory [40, 41].

#### Cognitive and behavioral skills

In order to measure each of the cognitive or behavioral skills for the five components to be examined in this trial in an efficient and valid manner, we have adopted the short versions based on the following established questionnaires. In order to measure the SM skill, we will use the five items that constitute the SM subscale of the original 17-item cognitive-behavioral self-monitoring scale developed by Tsuchida et al. [30]. The study confirmed the internal consistency reliability, factorial validity, and construct validity of the subscale. Each item is rated on a 4-point Likert scale from 0 = *Very untrue of me* through 3 = *Very true of me*, and the total score therefore ranges between 0 and 15.

The skills for CR will be measured with the six highest-loading items from the Competencies of Cognitive Therapy Scale, recently developed and validated by Strunk et al. [31]. Each item is rated from 0 = *Very untrue of me* through 3 = *Very true of me*, and the total score therefore ranges between 0 and 18. We ascertained the semantic equivalence between the Japanese version and its original English version through translation and back translation.

We will use the BA subscale of the Japanese Behavioral Activation for Depression Scale – Short Form, translated from the original English version [32] and validated in the Japanese population [33]. The Japanese version consists of five items, each rated from 0 = *Very untrue of me* through 3 = *Very true of me*.

AT will be measured by the self-assertion subscale of the Adult Social Skills Scale, developed by Aikawa et al. [34]. Its reliability, factorial validity, and construct validity have been established [34]. It consists of seven items, each rated between 0 = *Very untrue of me* through 3 = *Very true of me*.

The PS skill will be measured with the six highest-loading items of the approach avoidance style subscale of the Problem-Solving Inventory [35]. We developed its Japanese version through translation and back translation. The original study demonstrated the reliability, factorial validity, and construct validity for the original 32-item version. The reliability and validity of the abbreviated Japanese version need to be examined in this study. Each item is rated from 0 = *Very untrue of me* through 3 = *Very true of me*, and the total score ranges between 9 and 18.

#### CIDI

We will use the self-administered version of the Japanese WHO-CIDI 3.0 depression section [49, 50] to ascertain the diagnosis of a major depressive disorder according to DSM-IV in the past 12 months. The self-administered version has been shown to have good concordance with the clinical diagnosis of MDE [51] and to be reliable in a 1-year test–retest survey [44].

#### HPQ

We will use the three questions from the presenteeism subscale of the HPQ. The original study demonstrated its reliability and validity [36, 52]. The Japanese version of the HPQ has been used in the World Mental Health Survey in Japan [50].

### Participant timeline

**Fig. 2:**
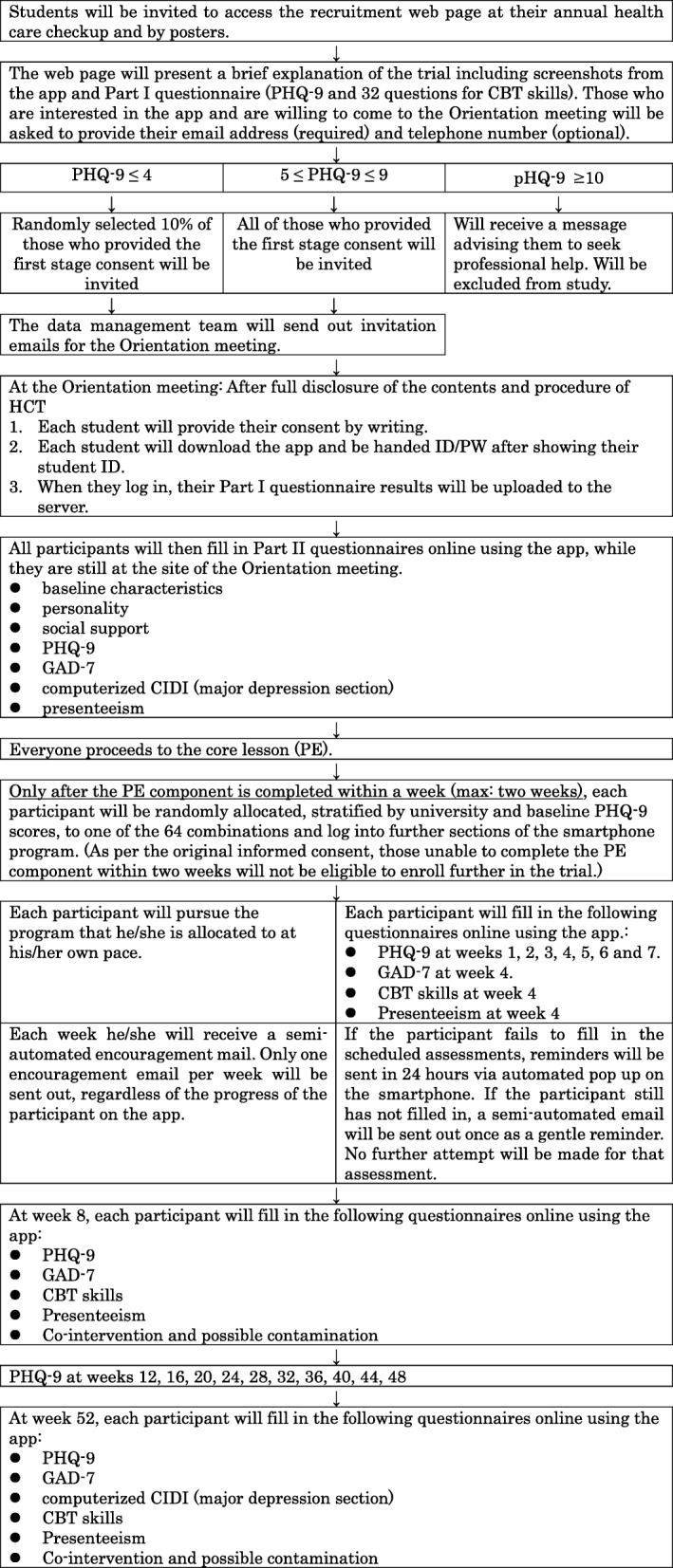
Enrollment, intervention, and assessment schedule

**Fig. 3:**
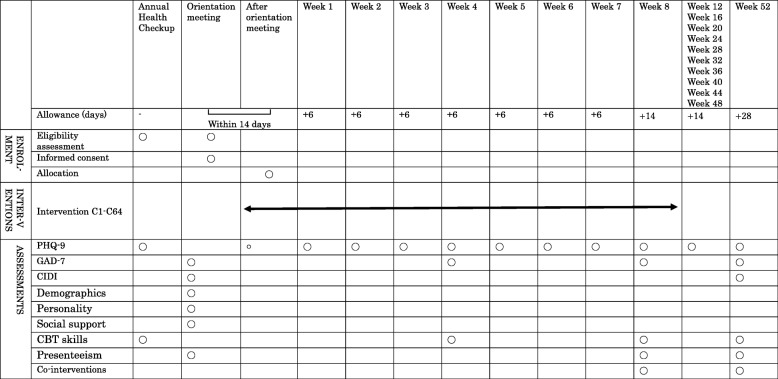
Enrollment, intervention, and assessment schedule

Figure 2 shows the participant timeline, and Fig. 3 shows the enrollment, intervention, and assessment schedule.

### Sample size

The study will be powered for analysis 1 (i.e., those scoring 5 or more on baseline PHQ-9). The following sample size is therefore for this stratum. The sample size will be reviewed after the pilot run in the first year, which will inform us of the possible participation rate among university students. All the power calculations have been conducted with FactorialPowerPlan SAS Macro (available for free download at http://methodology.psu.edu).

In order to detect an effect size (standardized mean difference) of 0.20 for an efficacious component and their interaction at alpha = 0.05 and beta = 0.10 in analysis of covariance (ANCOVA), we need a total sample size of 1051. To reach this total sample size, the sample size for each of 64 combinations needs to be 17 and the total sample size is therefore 17 × 64 = 1088.

The mixed-effects repeated-measures analysis with more than 5 measurement points, planned for this trial, is known to require 30–50% fewer participants for the same power than a pre-to-post-only assessment [53]. Because we expect less than 30% dropouts, we conservatively set the final target sample size of our study as that calculated by usual ANCOVA-type power calculation without repeated measures and without dropouts as above.

### Recruitment

In the first year of the study, we will conduct a pilot run aiming at enrolling 100–200 students at Kyoto University. This will inform us of the possible participation rate among the university students along with possible inadequacies and barriers in the protocol. In the second year of the study, depending on the findings from the pilot run, we will extend recruitment to other classes of undergraduate and graduate students at Kyoto University. In the third year of the study, depending on the recruitment so far, we will seek collaboration from several other universities in Japan. Recruitment from other universities may already be started in the second year.

### Assignment of interventions

#### Randomization and allocation concealment

The allocation will be stratified by university and by the baseline PHQ-9 score (4 or less vs 5 or more). We will use permuted block randomization in order to ensure balance in the number of subjects allocated to each combination, because in the fully factorial design, the imbalance in the allocated numbers among factors will reduce the statistical power of the study. The size of the block will be hidden to the study personnel, except for the statistician (HN) and the principal investigator (TAF), both of whom will have no role in the participant enrollment.

The statistician (HN) will generate the random allocation sequence using the SAS PROC PLAN (SAS Institute, Cary, NC, USA), which will be built into the server application. Each participant will then be automatically allocated to one of the combinations after he/she completes the PE component of the app within 2 weeks.

#### Blinding

Neither the participant nor the study personnel will be blinded to the intervention that each participant is receiving through the conduct of the trial. The assessment of all the primary and secondary outcomes is self-report by the participant and therefore not blinded. The statisticians will be blinded to the allocation through the statistical analyses by analyzing the datasets prepared by the study personnel in which all components are denoted only by a letter. The writing committee will review the statistical analysis report without knowledge of the identification of the components, which will be revealed only after the writing committee signs off the agreed-on statement of interpretation.

### Data collection, management, and analysis

#### Data collection methods

All the data will be collected via the smartphone app platform.

#### Plans to promote participant retention and complete follow-up

The outcome data will be collected even when the participant has not completed the allocated intervention, unless he/she has expressed a wish to completely withdraw from the study. When the participant fails to fill in the scheduled assessments, reminders will be sent in 24 hours and 48 hours via automated popup on the smartphone. If the participant still has not filled in the assessment, a semiautomated email will be sent once as a gentle reminder. No further attempt will be made for that assessment. The participants will receive modest compensation for the time it requires to fill in the questionnaires: 1000 yen when they complete week 4 assessment, 2000 yen when they complete week 8 assessment, and 2000 yen when they complete week 52 assessment.

#### Data management

A secure, web-based, password-protected database will be used to manage recruitment, eligibility assessment, randomization, scheduling and tracking, baseline and follow-up assessments, and delivery of the allocated interventions. All the assessment data will be checked automatically for integrity by this platform. The security of the data transfer between the app and the server will be guaranteed through the Secure Socket Layer (SSL).

### Statistical methods

All analyses will be performed on the intention-to-treat sample.

#### Analysis 1 main analyses at week 8

We will use the mixed-effects repeated-measures analysis to estimate the mean difference in change scores on the PHQ-9 for each component. The model will include random effects for subjects (as intercepts) and fixed effects of treatment (main effects and second-order interaction effects of the five components), visit (as categorical), and treatment-by-visit interaction, adjusted for university, age, sex, and baseline PHQ-9 scores. Each of the experimental factors will be coded at two levels (presence coded as + 1 and absence as − 1) using effect coding. The primary outcome is change in PHQ-9 scores from baseline to week 8. The estimated mean differences will be converted into standardized mean differences by using the root of the variance taken from the covariance matrix. No adjustment for multiple testing will be applied in estimation of statistical significance of the main and interaction effects in this model, and the conventional threshold for statistical significance (*p* < 0.05, two-sided) will be used, because in the optimization phase of the MOST framework, the emphasis is on making a decision about what components will make up the optimized intervention [20], and factorial designs usually evaluate multiple completely different interventions that could have been assessed in separate trials and, conventionally, the multiple hypotheses have been tested independently in these trial designs [54, 55]. We will use a similar model for GAD-7 and cognitive and behavioral skills at week 8. As sensitivity analyses, we will repeat the above-mentioned analyses for the whole sample, including both strata scoring 4 or less and 5 or more on baseline PHQ-9. The most efficacious and efficient combination(s) of smartphone CBT for university students will be proposed on the basis of all the above findings.

#### Analysis 1 secondary analyses

Exploratory analyses will be conducted to elucidate possible effect modification by the baseline variables on the efficacy of each component and of the proposed optimum combination(s) of components. The psychometric characteristics of the baseline questionnaires (internal consistency reliability, factor validity, and construct validity) will be ascertained. The ordering effect between BA and CR will be examined through comparison of the eight combinations in which CR comes before BA and the eight combinations in which BA comes before CR. The expected total sample size for this comparison is 272 (17 × 8 × 2). The comparison will have 70% power to detect an effect size of 0.3 at alpha = 0.05. Further exploratory analyses will be conducted on the mediator variables and use statistics of the smartphone CBT to examine the process of the therapy. Where repeated-measures analysis is not possible (e.g., cognitive behavioral skills), we will use multiple imputation for missing observations, where appropriate. The enduring effects of the 2-month intervention among those with elevated distress at baseline will be examined at week 52.

#### Analysis 2 main analyses at week 52

The primary outcome for analysis 2 is the incidence of a MDE by week 52 among all participants with or without elevated distress at baseline but without baseline MDE. We will use the mixed-effects logistic regression analysis to estimate the OR for each component. The model will include random effects for subjects (as intercepts) and fixed effects of treatment (main effects and second-order interaction effects of the five components), visit (as categorical) and treatment-by-visit interaction, university, age, sex, and baseline PHQ-9 scores.

An exploratory analysis will be conducted to compare the incidence of MDE between the proposed optimum combination(s) with the other combinations, which do not contain such components, through the time-to-event analysis (Kaplan-Meier survival analysis) among those who did not have an MDE at baseline. The influence of baseline characteristics on the time to event will be explored by use of Cox proportional hazards analysis if the proportional hazards assumption is met. This analysis is only exploratory, rather than confirmatory, because the efficacy of the proposed optimum combination(s) will be confounded by the correlations of depression scores and status between the 9-week outcomes, from which the proposed combination(s) are derived, and the 52-week outcomes, which are correlated with the outcome of this analysis.

Even when we include all participants with all baseline PHQ-9 scores, the power for analysis 2 may be low owing to the low event rates among the participants. The post hoc power calculation will be conducted for the expected 50% reduction in terms of OR or HR.

#### Interim analysis

No interim analysis is planned.

### Monitoring

#### Data monitoring

Data integrity will be monitored centrally, first through the built-in data check system on the server program and second by the data management team on a daily basis. The data management team will prepare the annual summary (number of participants entering the study, number of participants completing the study, and serious adverse events) to be presented to the data and safety monitoring board (DSMB) at 12 months, 24 months, and 36 months after the enrollment of the first participant. The DSMB will advise the steering committee as to the appropriateness of the study progress.

#### Harms

If elevated suicidal score is noted (total PHQ-9 score of 10 or more and “half or more of the time” or “almost daily” on item 9 of PHQ-9), the encouragement email will contain advice to seek help at the student health center and see a psychiatrist from the clinical management team who will judge whether any emergency intervention is necessary and whether the participation in the trial can be continued (participation itself can be continued after emergency intervention). All serious adverse events (defined as all deaths, life-threatening events, hospitalizations, enduring or conspicuous disabilities), whether they are related to the trial participation or not, will be handled according to the procedures set out by Kyoto University Hospital.

#### Auditing

Because the intervention can be classified as “minimally invasive intervention,” no formal auditing will be conducted.

### Ethics and dissemination

#### Research ethics approval

The study protocol was approved by the ethics committee of Kyoto University Graduate School of Medicine (C1357) and will be approved by the ethics committees of other collaborating universities.

#### Protocol amendments

Any amendments to the protocol will be submitted to the ethics committee of Kyoto University Graduate School of Medicine for approval. Once approved, they will be reported to all the study investigators and, where necessary, to the study participants. Further approval of the ethics committees of participating universities will also be sought.

#### Consent or assent

We will employ a two-stage consent procedure. At the time of the annual health checkup, participants will have access to the recruitment web page explaining the smartphone app and the trial, fill in part I of the questionnaire, and provide their contact details to receive an invitation to the orientation meeting. At the orientation meeting, the researchers from the student health center will obtain the fully informed written consent to participate in the trial on the smartphone app. Because all participants will be at least 18 years of age and the intervention is minimally invasive, no assent or consent from authorized surrogates is assumed for this study.

#### Confidentiality

Each participant will receive an identification number, and all the records will be managed using these identification numbers. The security of the data transfer between the app and the server will be guaranteed by SSL, and the data will be stored on the secure server. The data management team will download the data regularly and store them using a medium that is not connected to the internet and is kept in a locked drawer. Once the trial is completed, the data on the server will be erased permanently. The consent forms and the medium storing the downloaded data will be kept in the locked drawer in the student health center for 10 years after the publication of the primary findings. The de-identified, anonymized dataset will be uploaded to the UMIN-ICDR website (http://www.umin.ac.jp/icdr/index-j.html), and researchers approved by the steering committee will be able to have access to the dataset.

#### Access to data

All members of the steering committee will have full access to the final trial dataset.

#### Ancillary and posttrial care

All the participants receive the standard care as provided by the student health center of Kyoto University and by corresponding facilities in each participating university, both throughout the study and afterward.

#### Dissemination policy

The full protocol will be published in an academic journal in English. Its Japanese synopsis will be posted on the website of the student health center and the Department of Health Promotion and Human Behavior, Kyoto University Graduate School of Medicine/School of Public Health.

The study results will be disseminated to health care professionals and the public through presentations in academic meetings and publications in academic journals. The synopses of these reports will be posted on the home page mentioned above for their dissemination to trial participants.

Authorship of the planned primary and secondary publications will include all the members of the steering committee and others judged as appropriate by the steering committee. The order of the authors will be determined by the steering committee in consideration of the contributions of each member. After the publication of the primary findings, the de-identified and completely anonymized individual participant-level dataset will be posted on the UMIN-ICDR website (http://www.umin.ac.jp/icdr/index-j.html) for access by qualified researchers.

## Discussion

We have described the protocol for a MOST fully factorial trial aimed at optimizing the smartphone CBT for university students. The primary objective of this study is to estimate the specific efficacy of each of the five cognitive or behavioral components to reduce psychological distress within 2 months and thereby to propose the most efficacious and efficient combination of components for university students. The study has several secondary objectives, including the exploration of matching between treatments and individual characteristics and the examination of the prophylactic effect of smartphone CBT to prevent an MDE over the course of 12 months.

There will be many challenges in the conduct of this large-scale trial, including the recruitment of participants and their retention in the smartphone CBT and in the assessment. We have taken measures to meet these challenges as follows. First, Kyoto University, the primary study site, has over 22,000 undergraduate and graduate students, and we are anticipating participation of several similarly sized universities. Second, we have incorporated several trial design features to increase retention [56]: a run-in period before randomization so that only those who have shown preliminary adherence to the program are randomized; measuring the primary outcome at a relatively early time point (2 months); measuring all the outcomes online with several prompts built into the smartphone app; and offering appropriate monetary incentives, as approved by the ethics committee, for the completeness of data collection. Finally, the research team has a good track record in enrolling and following participants in previous trials [18, 57–59].

The theoretical strength of the proposed trial is its ability to estimate the specific main effect of a cognitive or behavioral component by comparing combinations including that component against those not including that component, all of which are equivalent in terms of treatment credibility, delivered attention, or therapist allegiance that may contribute to nonspecific effects. These findings can be expected to uniquely contribute to the theories of psychotherapy and of CBT [60].

The pragmatic advantage of the proposed trial comes from the MOST framework, which involves optimization trials using highly efficient experimental designs. This framework enables the investigator to identify the most efficacious and efficient package for a complex intervention. The resultant package of smartphone CBT can be efficiently delivered to a large number of students and attain the largest efficacy benefit for them. We also expect to move on to the evaluation phase of the MOST design in the future, in which we can not only evaluate this optimized intervention against treatment as usual but also collect further information to personalize the treatment to match the individual student’s characteristics and needs.

We will start the recruitment in 2018 and aim at completing the trial by 2021, and we expect to start publishing the results soon afterward.

### Trial status

Participant recruitment was started in April 2018 and is ongoing at the time of submission of this protocol paper.

## Abbreviations

ANCOVA: Analysis of covariance
AT: Assertion training
BA: Behavioral activation
CBT: Cognitive behavioral therapy
CIDI: Composite International Diagnostic Interview
CR: Cognitive restructuring
DSM: *Diagnostic and Statistical Manual of Mental Disorders*
DSMB: Data and Safety Monitoring Board
GAD-7: Generalized Anxiety Disorder-7
HPQ: Heath and Work Performance Questionnaire
MDE: Major depressive episode
MOST: Multiphase optimization strategy
PE: Psychoeducation
PHQ-9: Patient Health Questionnaire-9
PS: Problem solving
RCT: Randomized controlled trial
SM: Self-monitoring
SSL: Secure Socket Layer
WHO: World Health Organization
